# The consequences of sequence erosion in the evolution of recombination hotspots

**DOI:** 10.1098/rstb.2016.0462

**Published:** 2017-11-06

**Authors:** Irene Tiemann-Boege, Theresa Schwarz, Yasmin Striedner, Angelika Heissl

**Affiliations:** Institute of Biophysics, Johannes Kepler University, Linz, Gruberstraße 40, 4020 Linz, Austria

**Keywords:** recombination hotspots, PRDM9, double-strand breaks, binding motifs

## Abstract

Meiosis is initiated by a double-strand break (DSB) introduced in the DNA by a highly controlled process that is repaired by recombination. In many organisms, recombination occurs at specific and narrow regions of the genome, known as recombination hotspots, which overlap with regions enriched for DSBs. In recent years, it has been demonstrated that conversions and mutations resulting from the repair of DSBs lead to a rapid sequence evolution at recombination hotspots eroding target sites for DSBs. We still do not fully understand the effect of this erosion in the recombination activity, but evidence has shown that the binding of *trans*-acting factors like PRDM9 is affected. PRDM9 is a meiosis-specific, multi-domain protein that recognizes DNA target motifs by its zinc finger domain and directs DSBs to these target sites. Here we discuss the changes in affinity of PRDM9 to eroded recognition sequences, and explain how these changes in affinity of PRDM9 can affect recombination, leading sometimes to sterility in the context of hybrid crosses. We also present experimental data showing that DNA methylation reduces PRDM9 binding *in vitro*. Finally, we discuss PRDM9-independent hotspots, posing the question how these hotspots evolve and change with sequence erosion.

This article is part of the themed issue ‘Evolutionary causes and consequences of recombination rate variation in sexual organisms’.

## 1. Initiation of meiotic recombination by programmed double-strand breaks

Meiosis is a tightly regulated process ensuring the exchange of genetic material between homologous chromosomes, known as meiotic recombination. Genetic exchange between homologues first requires the formation of a programmed double-strand break (DSB) lesion in the DNA. During the repair of the DSB, the intermediate repair structure that results in a crossover (CO) also physically links the homologues (visible in cells as chiasmata), and ensures their proper segregation (reviewed in [1,2]). In addition, this exchange of genetic information is important to eliminate deleterious mutations from the genome, as was observed in asexual reproducing organisms or non-recombining regions of sexually reproducing organisms in which deleterious mutations accumulated at a higher rate [3].

Usually, DSBs are highly damaging to the cell. Hence, the formation of DSBs in meiosis is regulated by multi-step mechanisms directing their location. Thus, in many species DSBs are highly localized as was determined experimentally in yeast [4], mouse [5–7] and humans [8]. The downstream repair of DSBs is also localized and in most organisms, recombination is concentrated at discrete and narrow regions of the genome, 1–2 kb in size, known as recombination hotspots (reviewed in [9–11]). Meiotic recombination is not typically organized in hotspots or hotspots are not particularly strong in some species like worms (*Caenorhabditis elegans*) [12,13], *Drosophila melanogaster* [14] and the honeybee *Apis mellifera* [15]. Recombination hotspots (e.g. in humans) have been measured directly by sperm typing [16,17], pedigree analysis [18–21] or indirectly by comparing patterns of linkage disequilibrium (historical recombination hotspots) [22–24]. In humans, mice and budding yeast it was shown that DSBs overlap with centres of recombination [6–8,25–27] (also reviewed in [28]). Unlike budding yeast, DSBs in *Saccharomyces pombe* differ from the distribution of crossovers (reviewed in [28]). However, because our focus here is mainly on human and murine hotspots, for the purpose of simplicity DSB hotspots are handled as being equivalent to recombination hotspots and will be used interchangeably here.

We still do not fully understand the molecular factors determining the placement of DSBs, and thus the patterns of recombination hotspot locations and the meiotic recombination landscape. Mechanistically, a DSB occurs in a highly organized chromatin structure. During the first stages of meiotic prophase I, the chromatin undergoes substantial changes and is condensed into a tight structure formed by a series of tandem loops anchored by various proteins to axial elements at cohesion sites [29–31]. This structural chromosomal conformation is a key determinant for the placement of DSBs in leptotene. DSBs are preferentially introduced where several SPO11 accessory proteins, like the REC114-MEI4-MER2 complex (RMM complex), are located [31–33]. The mechanism of tethering the loop region with the axis for placing a DSB is proposed to be of high relevance for preventing inter-sister-chromatid repair [33]. The recruitment of DSBs to the axis has been postulated by Kleckner and co-workers in yeast as the ‘tethered loop–axis complex’ [31,32]. The hotspot is thought to be temporarily coupled to the axis by a bridge mechanism of SPP1 and MER2 that ensures the physical interaction of axis proteins and nucleosome depleted regions (NDRs) in the loops [34–36]. In the absence of SPP1, DSBs are reduced and redistributed [34,35].

When the axis proteins and the hotspot sequences on the loop interact, DSBs are introduced by a transesterification reaction of the topoisomerase-II like mechanism of SPO11 [25–27,37,38]. SPO11 shares similarities with the catalytic subunit A of the archaeal type II DNA topoisomerase (Topo VIA) [37]. Topo VI is a member of the type IIB enzyme family acting as a heterotetramer consisting out of two A and two B units leading to the relaxation of DNA supercoils by cutting and ligation steps [37]. Until recently, no Topo VIB subunit was identified in most eukaryotic cells, but *Arabidobsis thaliana* [39] and mice [40] have been shown to carry a variant of Topo VIB that is essential for meiotic DSB formation. In yeast, SPO11 is one of ten proteins forming four subcomplexes (SPO11-SKI8, REC102-REC104, REC114-MEI4-MER2 and MRE11-RAD50-XRS2), with also highly conserved homologues found in mammals (reviewed in [28]). Once DSBs are introduced, SPO11 is removed from the DSB site by an irreversible endonucleolytic cleavage, releasing small SPO11-bound oligos [41,42]. Subsequently, EXO1 mediates the 5′–3′-resection followed by the binding of replication protein A (RPA) to the newly formed 3′-single-stranded DNA tails (ssDNA). After the binding of the RecA family members DMC1 and RAD51, nucleoprotein filaments are formed which catalyse the invasion process into the homologue [43] forming recombination foci together with other factors (reviewed in [44]). After strand invasion of the free 3′-filaments and D-loop formation, the DSB can be repaired as a CO by the resolution of a double Holliday junction (dHJ) or as a non-crossover (NCO) by single-strand invasion that generates a non-reciprocal exchange of the sequence from one homologue to the other (reviewed in [9]).

## 2. Regulation of the recombination landscape by trans-factors

Factors controlling the position of hotspots have been elucidated only within the last two decades and many open questions regarding their relevance and function in recombination still remain open. In mammalian species, the recombination landscape is determined mainly by the *trans-*acting factor PRDM9; however, the role of PRDM9 in establishing hotspots has only been demonstrated in a few species. In taxa without a functional PRDM9, recombination is determined by *cis*-factors. Recombination is regulated by these *trans-* or *cis*-factors acting at a local scale; however, factors acting on a larger scale (with an independent effect from local factors) also play a role in the recombination landscape. These different factors, their role in recombination and their evolution will be discussed next with the main focus on PRDM9.

### (a) PRDM9: the main actor controlling the placement of DSBs

It has been demonstrated that PRDM9 specifies the location of recombination hotspots in mice and humans [5,8,45–47]. Also in great apes and cattle, PRDM9 dictates the recombination landscape [48–50]. Orthologues of PRDM9 have been identified in a large number of vertebrate species [51,52], but often these do not seem functional and lack several domains or even the full-length *Prdm9* (e.g. amphibians, birds, crocodiles and different fish lineages) [51–53]. Alternatively, PRDM9 has become dysfunctional by several premature stop codons as was observed in dogs [51,54].

PRDM9 is a meiosis-specific protein only expressed in male and female germ cells entering meiotic prophase I [55,56]. Specifically, it was shown in mice to be active from the pre-leptotene to mid-zygotene meiotic stage [57], in which the loop-structures and axial elements mentioned previously are formed. PRDM9 combines the functions of different domains to induce a sequence of events that potentially lead to the formation of a DSB. PRDM9 recognizes and binds specific DNA loci with its long zinc finger (ZnF) array [45,58–63]. The PR/SET domain marks the local neighbouring nucleosomes via H3K4- and H3K36-trimethylation [56,64,65]. The KRAB domain is involved in protein–protein interactions including CXXC1 (potentially important for the loop/axis interaction) [66,67]. There are some other domains within PRDM9, such as an SSXRD motif, a zinc knuckle and a single ZnF domain, whose functions are yet unknown (reviewed in [44]).

The functionality of each domain is still being elucidated, but it has been shown that the binding of the ZnF array of PRDM9 to a specific DNA sequence and the trimethylation of the surrounding nucleosomes by the PR/SET domain induces a reorganization of the surrounding chromatin structure around PRDM9 binding sites [7,58]. There is a positive correlation between the H3K4-trimethylation (H3K4me3) levels and NDRs, thus, it has been postulated that H3K4me3 is an important marker of meiotic recombination hotspots [58]. In fact, DSB hotspot centres are flanked by H3K4me3 tandem signals that decrease in intensity in both directions with distance to the hotspot centre [7,58]. These H3K4me3 pattern around the majority of the hotspot centres is asymmetric and independent of the orientation of the PRDM9 recognition motif [7]. SPO11 preferentially cleaves at central NDRs, but is also able to target flanking NDRs, albeit less frequently [7]. The role of the H3K4me3 epigenetic mark is not yet fully understood. Functionally, this mark creates an open chromatin structure and has been observed to be constitutively high in nucleosomes flanking NDRs, as found in gene promoters in budding yeast [36]. However, the presence of this mark is not sufficient for the formation of DSBs. In yeast, it was reported that the interaction of H3K4me3 with different components of the recombination initiation machinery, like Spp1, is also required [34,35]. Similarly, it was proposed recently that this epigenetic mark is also important together with the combined interaction of the different domains of PRDM9 for directing the activity of the recombination initiation machinery. Specifically, PRDM9 interacts (maybe indirectly) with components of the synaptonemal complex, as well as with cohesins, like REC8 in a complex with helper proteins (such as EWSR1 and CXXC1) [66,67]. The exact role of these PRDM9 interactions has not been elucidated yet, but it has been hypothesized that PRDM9 acts in concert with these proteins (such as the Spp1 orthologue CXXC1) to tether the bound hotspot DNA to the chromatin axis for the initiation of recombination via SPO11, potentially through the double interaction of CXXC1 with PRDM9 and H3K4me3 [66]. Moreover, kinetic studies have revealed that PRDM9 forms a highly stable complex with its binding target that does not dissociate for many hours [63]. It is possible that the formation of such a stable complex is important for the interaction of the different PRDM9 domains within the hotspot until DSBs are introduced. It is still unclear whether PRDM9 is removed from the DNA before SPO11 cleavage or if SPO11 can cut PRDM9-bound DNA [7].

### (b) How does PRDM9 determine the positioning of hotspots

**Figure 1.**
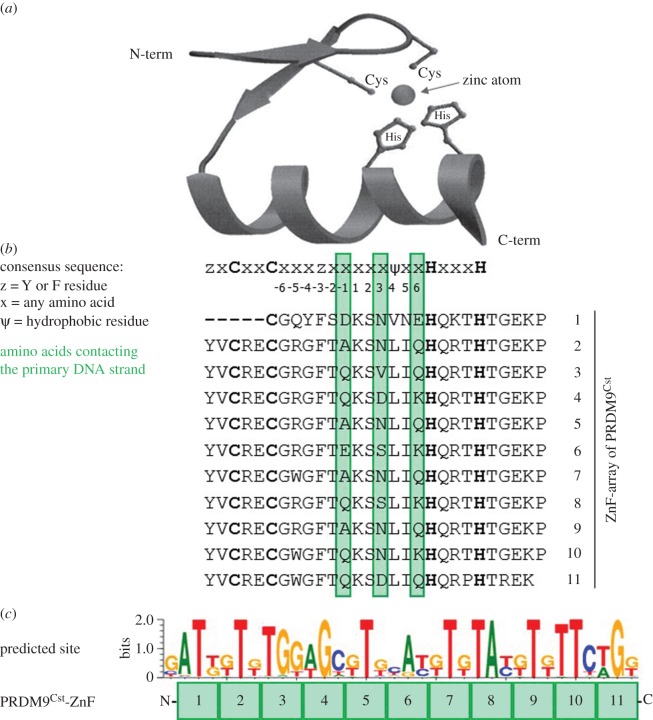
(*a*) Three-dimensional structure of a representative single C2H2-type ZnF, harbouring two antiparallel beta-sheets and an alpha helix, which contains the DNA-binding amino acids, as well as a zinc atom coordinated by two cysteines and two histidines (adapted from Wolfe *et al.* [68]). (*b*) Consensus sequence of a C2H2-type ZnF [68] aligned with the ZnF array of PRDM9^Cst^. The DNA-contacting residues (at positions −1, 3 and 6) are highlighted in green. The zinc coordinating amino acids are shown in bold letters. (*c*) Predicted binding site of the 11 PRDM9^Cst^-ZnFs using the Persikov algorithm (polynomial SVM settings) [69] aligned to the respective ZnFs. Shown is the reverse DNA strand in 5′–3′ direction (the amino acids at positions −1, 3 and 6 contact the forward strand).

Understanding what factors drive the binding of the ZnF array to a specific nucleotide sequence is key to elucidate the positioning of DSBs, and thus the recombination landscapes shaped by PRDM9. On a molecular level, the intramolecular forces acting between the amino acids of the ZnF array and the DNA determine the specificity of this protein-DNA interaction. Extensive studies of C2H2-type ZnF proteins have revealed a particular DNA-binding pattern of this protein class, postulated as the canonical binding model (reviewed in [68]). According to this model, each individual ZnF interacts with four nucleotides in the DNA (reviewed in [68]). More specifically, amino acids at positions −1, 3 and 6 of the alpha helix (in respect to the inner Zn-coordinating cysteine and histidine) interact with three consecutive nucleotides on the primary DNA strand, while the amino acid at position 2 binds to the complementary strand (figure 1). Based on the chemical properties of the amino acids present at these key positions, it is therefore possible to predict the theoretically most favourable nucleotide sequence for a particular ZnF protein [69–72].

Evidence from mice and humans have shown that the type and number of ZnFs in PRDM9 have a strong influence in the hotspot landscape [5,6,60,73–77]. Hundreds of different PRDM9 alleles, which differ both in the number of ZnF repeats and their identity (DNA-contacting residues) have been identified, and the numbers keep increasing as more sequencing data becomes available [19,45,47,73,74,78,79]. Each PRDM9 allele recognizes its own specific DNA motif, and thus different PRDM9 alleles account for distinct hotspot landscapes in the population [73,74,77].

For example, in mice, the hotspot overlap was analysed between different variants of murine PRDM9 [5,6,76]. Congenic strains of mice with different PRDM9 alleles (strains B10.S-H2t4/(9R)/J and B10.F-H2pb1/(13R)/J, shortly 9R and 13R with 12 and 11 ZnF repeats, respectively) showed hardly an overlap of hotspots, yet closely related strains sharing the same PRDM9 allele (e.g. 9R and C57BL/6 J, also known as B6) shared 98% of the hotspots [5,6]. In F_1_ hybrids from crosses between the six mouse strains 13R, B6, C3H (C3H/HeJ strain; all three from *Mus musculus domesticus* origin), Cst (CAST/EiJ strain, *M. m. castaneus* origin), MOL (MOLF/EiJ strain; *M. m. molossinus* origin) and PWD (PWD/PhJ strain; *M. m. musculus* origin) only about 1.1% of hotspots in average were shared between the strains with different PRDM9 alleles. However, those PRDM9 alleles with the most similar ZnF arrays, PRDM9^B6^ and PRDM9^C3H^, showed an overlap of approximately 30% of hotspots in the F_1_ hybrid crosses [75]. A further experiment in a hybrid cross between strains with different genetic backgrounds (*M. m. castaneus* and *M. m. domesticus*, Cst and C57BL/6 J-PRDM9^Cst-KI^/Kpgn, for simplicity B6^Cst-KI^, respectively) with the latter being a knock-in strain in which the *Prdm9* allele was replaced by the one from the Cst strain, showed that the introduction of the foreign *Prdm9* allele led to the activation of Cst-specific hotspots in the *M. m. domesticus* genetic background [76]. This strongly indicates that the hotspot landscape is determined mainly by the nature of the ZnF array of PRDM9. Finally, *in vitro* studies of different murine PRDM9 alleles confirmed a specific binding of mouse PRDM9 variants to their predicted motifs [58,60,62].

Also in humans, hotspots active in individuals carrying the PRDM9^A^ allele (very common in Europeans), were inactive in individuals with the PRDM9^C^ allele (more common in Africans) and vice versa [73,74]. Similarly, the comparison of DSB maps in five males, two homozygous for the allele PRDM9^A^ and three heterozygous for the A and the less frequent allele B or C, showed a large overlap of hotspots between the almost identical alleles A and B, but only a partial overlap with AC heterozygotes [8]. PRDM9 allele A versus C varies in the number and type of ZnFs contacting the DNA [8,73], with very different DNA motifs predicted and enriched at hotspots specific for Europeans or Africans [46,77]. It was also shown that the human allele PRDM9^C^ recognizes a different 17 bp motif enriched exclusively at hotspots from the African population [8,73,74,77]. Moreover, it has been shown that allele PRDM9^A^ specifically binds its motif *in vitro* [45,61], but not to the DNA sequence recognized by a different allele (PRDM9^I^) [45]. Even small variations in the amino acid sequence of the ZnF array or variations not predicted to affect DNA binding can trigger the appearance or disappearance of a hotspot, as was observed for the MSTM1a hotspot, which was activated by a single Lys>Glu substitution in PRDM9 [73].

It seems clear that the ZnF array of PRDM9 defines the recombination landscape in mice and humans, turning hotspot on or off. Does this also mean that the presence of a DNA motif recognized by the ZnF array is necessary or sufficient to create a hotspot? The analysis of human historical, genome-wide recombination hotspots inferred from patterns of linkage-disequilibrium of the HapMap Phase II data showed that one or more copies of the 13 bp PRDM9 binding site (Myers motif) co-localizes with approximately 40% of the hotspots (especially in the context of the THE1A transposon in which 73% of the hotspots contained the motif [24]). This measured percentage of hotspots containing the PRDM9 motif could be related to the resolution of the hotspots and not to the importance of the PRDM9 motif in hotspot specification. Indeed, higher resolution hotspots obtained by mapping double-strand breaks genome-wide with ChIP-seq (chromatin immunoprecipitation followed by sequencing) experiments, showed that approximately 70% of functional PRDM9 binding sites in humans lie within 250 nt of the experimentally determined DSB centres [8].

However, the DNA recognition motifs recognized by PRDM9 are neither necessary nor sufficient to specify hotspots (reviewed in [80]). The Myers or the murine motifs are found more often outside than inside hotspots [5,46], and some human hotspots (historical or DSB hotspots determined by sperm typing) do not have a motif [73,74]. Other difficult to explain observations about PRDM9 binding have been made in mice. For example, PRDM9^Cst^ activates multiple hotspots that do not share an obvious consensus sequence (e.g. *Psmb9*, *Hlx1* and *Esrrg-1*) [59]. For this particular PRDM9 allele, the DNA-contacting amino acid compositions of ZnFs 2, 5, 7 and 9 contact different nucleotides of the analysed sequences. Moreover, the recognition of a DNA sequence by a particular ZnF can change depending on its location in the array and neighbouring ZnFs [59,61]. The absence of a motif in a hotspot might be explained by the binding plasticity of PRDM9. It was shown in two different studies that a subset of ZnFs in the array can already bind specifically to a target sequence, as was shown for PRDM9^A^ [61] and PRDM9^Cst^ [63]. Interestingly, when replacing the DNA by different unspecific nucleotides, the binding strength of two different subsets was similar, suggesting that different ZnFs of the array can engage in specific or unspecific interactions interchangeably [63]. However, the affinity of PRDM9 to the DNA was considerably increased when all ZnFs of the array contacted the DNA and was enhanced even further when the target DNA contained neighbouring flanking regions [63]. This plasticity of the PRDM9-ZnF could explain why the canonical binding predictions based on the amino acid sequence of the ZnF array do not always match the DNA sequences found in recombination hotspots or why the presence of a canonical binding motif is not necessary in an active hotspot.

### (c) DNA methylation at target sites also affects PRDM9 binding

In addition to the well-described sequence recognition of naked DNA motifs by PRDM9, epigenetic modifications such as DNA methylation of cytosine at a CpG dinucleotide might also influence PRDM9 recognition and binding, given the different chemical structure of 5-methyl cytosine (5-meC) compared to cytosine. Different from plants, more than 98% of the cytosine methylation occurs within the context of a CpG in mammals [81], except for embryonic stem cells in which a higher proportion of DNA methylation occurs outside CpGs [81,82]. Thus, DNA methylation could be an important parameter influencing PRDM9 binding, especially for PRDM9 alleles that recognize motifs harbouring CpG sites. Such is the case for the common human PRDM9^A^ allele, which recognizes the degenerate Myers motif (CCnCCnTnnCCnC; with ‘n’ defined as a nucleotide with no influence in the affinity according to the position weight matrix) [24] with three potential CpG sites within 13 bp (23% potential methylation) and its extended *in silico* predicted motif nnnnnCnnnACnACnAnnAnnAnCCnCCnTaaCCnCCnnn, with seven potential CpG sites in 40 bp (17.5% potential methylation) [46]. Note that the Myers motif is enriched at the THE1 transposable element, which is common in GC-rich regions that are usually methylated [23,83]. Not only the Myers motif recognized by human PRDM9^A^ and PRDM9^B^, but also the sequence motifs for PRDM9^C^ (CCnCnnTnnnCnTnnCC) found in the African population [74,77] and the predicted binding site for the rare PRDM9^I^ (nGnnCnnnnCnnCnnnnnnnnnCCGCnGTnnnCGTnGTnGTnnCCGn) [45] contain putative CpG sites that might be targets for DNA methylation. In comparison, predicted binding motifs for primate PRDM9 alleles like the chimpanzee (AATTnnAnTCnTCC), gorilla (CCnAnnCCTC), macaque (GACGAnA) and simia (GnTGCTC) [79], as well as the recently identified sequence motifs for certain murine PRDM9 alleles [75], show less number of putative CpG sites.

Nevertheless, given the importance of the Myers motif in the context of human hotspots, we investigated the role of 5-meC on PRDM9 binding using an *in vitro* binding experiment. Given our lack of a recombinant human PRDM9^A^, we decided to perform this experiment using our well-characterized murine PRDM9^Cst^, for which we have collected extensive binding data and controls for the *Hlx1* DNA sequence [63]. However, the binding motif recognized by PRDM9^Cst^ does not contain putative CpG sites, thus in order to keep all the parameters the same except for cytosine methylation, we replaced different cytosines in the *Hlx1* sequence by 5-meC (in this case outside a CpG context). We hypothesize that our results can still be generalized to other PRDM9 alleles with CpGs in their motifs in terms of their binding to methylated DNA.

**Figure 2.**
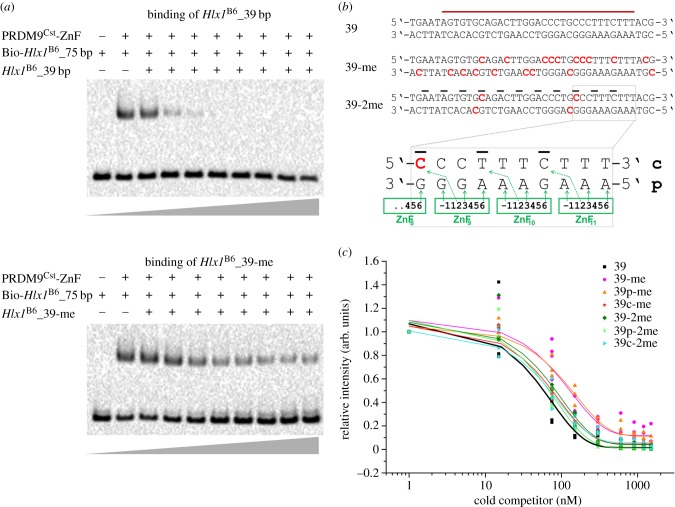
The influence of DNA methylation in PRDM9 binding. (*a*) EMSA competition assays were performed by incubating 250 nM murine PRDM9^Cst^-ZnF with 15 nM hot *Hlx1*^B6^ DNA (75 bp in length) and increasing concentrations of an unlabelled (cold) 39 bp *Hlx1*^B6^ DNA fragment, carrying different levels of methylation. Representative EMSAs are shown for the cold competitors with 39 bp and the fully methylated 39 bp fragment 39-me. (*b*) An overview of the 39 bp cold competitor DNA sequences with different levels of methylation is shown. The red-coloured letters show the methylated cytosines (5-meC) on the respective strands. The red bar indicates the *Hlx1*^B6^ minimal binding site [59]. The black bars indicate the nucleotides that interact with position 2 of the zinc finger repeats (according to the prevalent canonical binding model of C2H2-type ZnF proteins as described in [68,70]). The magnified area shows a detailed representation of the expected amino acid-nucleotide contacts on both the primary and complementary strand. The green arrows indicate the DNA-contacting amino acids at positions −1, 2, 3 and 6 of the α-helix of each ZnF domain. (*c*) Plot representing the relative intensity of the complex (shifted band) with and without cold competitor, as a function of increasing amounts of competitor (0–100× excess). The relative intensities are plotted against the concentration of the cold competitor in a semi-logarithmic graph. The DNA sequences were either fully methylated (39-me; methylation of all cytosines) or partially methylated (39-2me; methylation of only two cytosines) on either the primary (p) or the complementary (c) DNA strand.

For our binding study, we produced several DNA-fragments containing 5-meC in the *Hlx1* sequence and tested their binding to the PRDM9^Cst^-ZnF in an EMSA (Electrophoretic Mobility Shift Assay) competition experiment (figure 2). The PRDM9 lysate was incubated with constant amounts of hot (biotinylated), unmethylated 75 bp *Hlx1*^B6^ and increasing concentrations of cold (non-biotinylated) 39 bp *Hlx1*^B6^ that varied in methylation levels (figure 2). Differences in the shifted band (complex) indicate the strength of the competition with the hot, unmethylated DNA (figure 2*a*). The experiment was designed to follow the canonical binding model of C2H2 ZnFs showing differences in the number of amino acid–DNA interactions between the primary and the complementary strand, as discussed previously [68,70]. In short, each single ZnF of the C-terminus of the protein binds a nucleotide triplet of the 5′-end on the primary DNA strand (figure 2*b*) and only 1 nt on the complementary strand from the next nucleotide triplet [68,70]. Thus, we also tested the effect of methylation on the primary DNA strand versus the complementary strand. The DNA was either methylated on both or only one strand, indicated as the primary (p) or complementary (c) strand, respectively (figure 2*b*). In one configuration, all 9 or 10 cytosines of the 39 bp *Hlx1* binding site (within the 23% methylation potentially found in the Myers motif) were replaced by 5-meC (full methylation), and in the other configuration only two out of nine or 10 cytosines (partial methylation; 5% methylation) were replaced. These two 5-meC were placed at positions expected to be directly contacted by the amino acids of ZnF3 and ZnF8, given that these two ZnFs of the PRDM9^Cst^ array were shown to be especially important for conferring binding specificity [63].

We observed a significantly weaker competition (*p* < 0.0016; generalized least-squares model corrected for non-homogeneous variances and auto-correlation; see details in [63]) with the fully methylated DNA fragment (methylation of both strands; 39-me) or just the primary strand contacting the PRDM9-ZnF array (39p-me). By contrast, for the experiment in which only the complement strand was methylated (39c-me), or for the partially methylated DNA (39-2me, 39p-2me and 39c-2me), no significant difference was observed compared with non-methylated DNA, suggesting that heavily methylated DNA, especially in the primary strand, reduces PRDM9 binding *in vitro*. In conclusion, DNA methylation might reduce the binding affinity of PRDM9 to motifs with at least approximately 20% putative methylated CpG sites. Consistent with our results showing that low cytosine methylation does not affect PRDM9 binding, was the observation that in a study of a truncated version of the human ZnF array (ZnF 8–12), the methylation of one CpG site in a 21 bp sequence did not influence the binding affinity of this ZnF array [61]. The inhibitory effect of methylation in PRDM9 binding might be amplified by proteins that target methylated CpGs like MeCP2, competing with PRDM9 for binding. Alternatively, the binding of proteins like CXXC1, which could interact with PRDM9 and have a high affinity for unmethylated DNA, also might be compromised by DNA methylation [66].

## 3. DSBs lead to a rapid sequence evolution at hotspots by mutations and conversion events

Different lines of evidence have shown that the sequence composition at recombination hotspots is rapidly evolving. Based on sequence comparisons, a higher genetic diversity among humans was observed at recombination hotspots. Specifically, an elevated human–chimp divergence was found in regions of high recombination (reviewed by [84–89]). In addition, a higher number of substitutions has been correlated with recombination in other eukaryotes (reviewed in [90–92]). Moreover, target motifs recognized by the recombination machinery that are recurrently targeted for DSBs are more eroded in humans than the orthologous sequences in chimpanzees [46], and more substitutions have been observed in the near vicinity of these target motifs [93].

One process recognized as a major evolutionary force, reshaping the genomic nucleotide landscape at recombination hotspots, has been the mutagenic activity of recombination. More mutations were measured experimentally in human sperm in a DNA region with a CO than without a recombination event [94]. The overall mutation rates for CO (approx. 9.3 × 10^−7^) were almost two orders of magnitude higher [94] than the average mutation rate reported for the human germline (approx. 1.2 × 10^−8^) [95,96]. Similarly, meiotic yeast cells showed approximately five to 20 times more mutations in reporter genes than mitotic cells [97]. The strong mutational bias at CpG sites compared to non-CpGs observed in human crossovers suggested that mutations in crossovers are likely the product of deamination of methylated cytosines [94]. Single-stranded DNA, observed in resected 3′-ends or single-stranded filaments (covered by RAD51 and DMC1) formed during the repair of DSBs, is approximately 1000 times more susceptible for deamination, if not protected by the complementary strand ([98] and references therein). Moreover, the repair of the resulting DNA lesion is affected within a single-strand context: thymine (the deamination product of 5-meC) cannot be recognized as a DNA lesion by the repair machinery in single-stranded DNA; whereas, uracil (deaminated cytosine)—a foreign base in the DNA—can be identified and removed. The mean resection zone at programmed meiotic DSBs forming single-stranded DNA averages approximately 0.75–1 kb in length in mouse [7], which is congruent with the zone in which GC to AT transition mutations were observed [94].

A second process reshaping the genomic nucleotide landscape at recombination hotspots is the biased transmission of alleles by gene conversion. The repair of DSBs can lead to a biased nucleotide composition at recombination hotspots by different gene conversion mechanisms, which is the non-reciprocal exchange of DNA stretches of one chromosome to the other. As long as both alleles on the reciprocals have the same probability for gene conversion, there will not be a biased allelic transmission. However, if one allele has a higher probability for transmission over the other, its frequency in the gamete pool increases. A higher transmission frequency of one allele over the other via biased gene conversion (BGC) gives rise to an evolutionary advantage of fixation at the acceptor locus (reviewed in [91]).

One well-known BGC mechanism is GC-biased gene conversion (gBGC), in which the transmission of GC over AT variants are favoured during recombination. Indirect evidence for gBGC comes from sequence comparisons which observed an overall enrichment of GC content with recombination, as well as, a bias for the fixation of GC (strong) over AT alleles (weak) at recombination hotspots in humans [86], chimpanzees [50,86], mice [99] and other metazoans [100]. In budding yeast, BGC tracks were observed experimentally in a four tetrad analysis within approximately 1–2 kb in length of the DSB region [101,102]. The origin of gBGC is still not understood and may differ between organisms. In budding yeast, the preferential replacement of GC over AT observed over longer patches supports the role of mismatch repair driving gBGC in this particular case [101,102]. Also in humans, the over-transmission of GC over AT polymorphisms was observed experimentally in crossovers [21,94], as well as NCO products [21,103,104]. However, in humans, the conversion tracts spanned only one to two polymorphisms within a 0.1–0.3 kb polymorphic region [94,103] compared with the on average 0.5–2 kb tracks or longer observed in yeast [102], suggesting a contribution of a repair system that produces short conversion tracks, such as short patch repair by base excision repair rather than mismatch repair [94]. It has been postulated that gBGC is an adaptation to reduce the mutational load of recombination [91,94,105]: gBGC enriches the GC content in regions with high recombination; whereas, mutations occurring during the DSB repair are biased towards AT [94]. However, it was estimated, based on experimental mutation and gBGC rates at a recombination hotspot, that gBGC has a stronger effect than mutagenesis over longer periods of evolution [94], which might explain why recombination hotspots are GC-rich [86,91].

Another mechanism leading to BGC events is meiotic initiation bias. By comparing the CO junctions of both reciprocal recombination products measured by sperm typing, Jeffreys and colleagues observed a transmission distortion that involved polymorphisms within a 100–200 bp region centred at the peak of the hotspot [106–108]. This distortion, known as meiotic initiation bias, is caused by the preferential placement of a DSB on one of the two homologues. During the DSB repair, the intact homologue serves as a template to repair the homologue with the DSB leading to an over-transmission of the alleles from the unbroken to the broken homologue (reviewed in [9]). In contrast with gBGC, this type of conversion is independent of the GC content of the polymorphisms. Instead, the direction of the transmission depends on the DSB-promoting allele at the target site recognized by the initiation recombination machinery, like PRDM9 [46]. The allele that binds the recombination machinery best, will be a target for a DSB and will get lost during the repair and resolution of the recombination event [46]. A loss in programmed DSBs also eliminates the recombination hotspot, given that recombination requires DSBs for its initiation. In other words, hotspots have a tendency to self-destruct through the systematic over-transmission of alleles downregulating recombination. This has been known as the hotspot-paradox, in which a DSB-promoting allele drives its own loss [106,109–111].

The rapid sequence erosion by mutations and gene conversion at DSB target sites has posed the question, together with the hotspot-paradox, about the effect of the sequence evolution on the tight control and placement of DSBs, and thus, hotspot activity. Although, we still do not fully know all the players, important advances were made in the past years about the role of *trans*-acting factors like PRDM9 in relocating hotspots to less eroded or ‘virgin’ sites, defined as sequences that have not got in contact with a particular PRDM9 allele, which will be discussed next.

## 4. What is the effect of eroded target motifs on recombination hotspots?

### (a) Sequence erosion at target sites affects PRDM9 binding

Changes in the ZnF array have a strong influence in hotspot usage, turning hotspots on or off and targeting different regions of the genome [5,60,73,74]. What about changes in the DNA recognition sequence? Does the sequence erosion at the binding motifs affect PRDM9 recognition and thus hotspot specification? Human and murine high-resolution DSB maps showed that mismatches to a consensus motif reduce the strength of the hotspot and modulate the intensity of the hotspot [6,8]. In fact, a single-nucleotide polymorphism (SNP) occurring at a ‘high score’ base (i.e. a base that is important for PRDM9 binding specificity) within a motif can completely disrupt hotspot activity [8]. Generally, changes in a ‘motif score’ were positively correlated with changes in hotspot strength genome-wide [8]. Also in high-resolution murine DSB maps, the match to the motif was correlated with the strength of the hotspot [5,6]. Similar observations were also made in human sperm typing studies [106,108] and mouse crosses [75,76,112]. A substitution within the recognition motif can affect hotspot activity. These polymorphisms lead to the preferential binding of the recombination initiation machinery to only one of the heterozygous homologues targeted for a DSB, observed as an initiation bias, as discussed in the previous section.

*In vitro* studies have also observed a change in the binding of PRDM9 to recognition sequences with different nucleotide substitutions [59,60,76]. For example, 2 nt within a 60 bp sequence of the *Psmb9* hotspot or 4 nt in a 41 bp sequence of the *Hlx1* hotspot significantly impaired the binding of PRDM9^Cst^ (found in *Mus musculus castaneus*) [60]. However, not all nucleotide substitutions in the recognition motif have the same effect on PRDM9 binding. The examination of the *in vitro* PRDM9^Cst^ binding with a high-affinity hotspot sequence (*Hlx1*), in which all possible single nucleotides were replaced one at a time, showed that certain nucleotide substitutions had a stronger disrupting effect in PRDM9 binding than others [59]. For example, single-nucleotide substitutions contacting the N-terminal ZnFs (especially ZnFs 4–7) exhibited a stronger disrupting effect in the PRDM9–DNA interaction than substitutions contacting fingers at the C-terminal end [59].

Recently, it was shown that certain nucleotide substitutions in the motif directly affect the binding affinity of PRDM9 [61,63]. Striedner *et al*. showed that the *in vitro* dissociation constant (*K*_D_) of PRDM9^Cst^ from *M. m. castaneus* for its own *Hlx1* recognition sequence was five times lower compared with the orthologous sequence present in *M. m. domesticus* [63]. These target sequences differ by three SNPs and one in-del, and are an example of the effect of sequence erosion of motifs in active recombination hotspots that affect PRDM9 binding. Similar observations were made for the human PRDM9^A^ variant, where a single-nucleotide substitution within the recognition site (changing a ‘high score’ base in the Myers motif in the THE1B retrotransposon) resulted in approximately fivefold reduced affinity [61]. At the same time, this nucleotide substitution lead to approximately twofold increased affinity of the PRDM9^L20^ allele [61].

### (b) Rapid evolution of PRDM9 relocates hotspots to ‘virgin’ targets

Given that sequence erosion directly affects the affinity for PRDM9, less eroded or virgin sequences (sequences that had no contact with PRDM9 and thus were not a recurrent target for DSBs resulting in motif erosion) bind PRDM9 better compared with eroded sequences. Indeed, the analysis of the H3K4me3 patterns near the binding motif of PRDM9 in a hybrid cross between two evolutionary distant murine subspecies (*M. m. domesticus* versus *M. m. castaneus* expressing PRDM9^B6^ and PRDM9^Cst^, respectively) showed the preferential H3K4me3 (a proxy for PRDM9 binding, as shown in [58,112]) to the less eroded chromosome of the other subspecies. That is, PRDM9-driven H3K4me3 around PRDM9^Cst^ sequence motifs was observed more often in *M. m. domesticus* derived chromosomes than on self-chromosomes and vice versa [76]. *In vitro* binding studies with EMSA, testing a few hotspot sites, confirmed that PRDM9^B6^ indeed binds preferentially to the less eroded sequence of the *M. m. castaneus* [76]. An even more extreme bias in the preferential binding of PRDM9 (measured by DSB formation and H3K4me3) to the less eroded chromosome was observed in a different hybrid cross between the strains PWD/Ph and C57BL/6 J (PWD × B6) [112]. Also in another study, a similar phenomenon was described in different crosses of four major mouse strains and their F_1_ hybrid crosses (*M. M. musculus*, *M. m. domesticus*, *M. m. castaneus* and *M. m. molossinus*), with a total of six different *Prdm9* alleles [75]. Mapping the DSB sites in these crosses discovered up to 35% novel DSB hotspots (hotspots present in the F_1_ offspring but absent in the two parental strains), of which 79% of such novel hotspots showed DSB formation strongly biased to the ‘non-self’ parental chromosome. Moreover, in most of these novel hotspots, a polymorphism was found to improve the PRDM9 binding site compared with the parental chromosome [75].

Comparison of the ZnF array of PRDM9 in different mammalian species, primates or humans, and with other ZnF proteins of the C2H2-type, revealed a high level of divergence and an exceptionally rapid evolution of PRDM9 [78,79,113,114]. The rapid evolution of orthologous *Prdm9* genes between species was not only shown in primates [51,78], but also in bovids [115], equids [116], goats and sheep [117], as well as in rodents. This rapid evolution occurs mainly on residues contacting the DNA (figure 1) suggesting that these changes might be driven by concerted evolution and positive selection [51]. The rapid evolutionary changes in PRDM9 have also been observed in several metazoan lineages including the sea anemone *Nematostella vectensis* and the gastropod snail *Lottia gigantean* [51].

Given that nucleotide changes in target sequences directly affect the affinity of PRDM9 and thus hotspot activity, it has been postulated that the astonishing diversity of the ZnF array of PRDM9 is an adaptation to relocate recombination hotspots to less eroded sites and restore the recombination activity [45,46,51,118]. The hypothesis of a trans-acting factor, like PRDM9, giving birth to new hotspots has been also proposed for solving the hotspot-paradox [110,119]. The diversity of PRDM9 is also the fortunate outcome of the sequence properties of its locus. The ZnF array of PRDM9 is coded by a highly dynamic sequence with a series of tandem repeats, 84 bp in length (28 amino acids), known as minisatellites (figure 1). An intrinsic property of minisatellites is their high instability, rapidly gaining, loosing or replacing repeats as a by-product of mitotic and meiotic recombination involving both inter- or intra-allelic non-reciprocal conversion events. The instability of the repeat sequence coding for PRDM9 substantially remodels the ZnF array in a fairly short evolutionary time and counteracts the sequence erosion at hotspots by relocating recombination to virgin sequences [118]. Intriguingly, PRDM9 seems to drive its own evolution, with certain PRDM9 variants creating more de novo variants in the germline than others, with the most unstable PRDM9 alleles predicted to quickly being eliminated and not contributing to the pool of PRDM9 variability [118].

### (c) The preferential binding to ‘virgin’ target sequences leads to hotspot asymmetry

The rapidly evolving ZnFs of PRDM9 have different recognition sequences. Thus, different PRDM9 alleles result in different recombination landscapes, even between closely related species (e.g. human and chimp) [50,120] and populations (e.g. Africans versus Europeans) [8,73,74,77]. When a new PRDM9 allele is introduced into a different genetic background (e.g. hybrid cross), new sites in the genome are targeted for programmed DSBs creating novel hotspots [75,76,112] because PRDM9 has a higher affinity for the less eroded target sequences that have not encountered that PRDM9 allele [63]. In hybrid crosses that are heterozygous for both native and virgin sequences, it has been observed that mainly the virgin chromosomes are targeted for DSBs, resulting in an asymmetric distribution of hotspots between homologues. That is, hotspots are located at different target motifs in each homologue, respectively [75,112]. This asymmetry was analysed in full detail in F_1_ hybrids of PWD × B6 and reciprocal crosses that carried heterozygous PWD and B6 chromosomes and two different PRDM9 alleles (PRDM9^Pwd^ and PRDM9^B6^). The recombination activity was measured by DMC1 ChIP-seq signals (representing DSB genome-wide maps), and genome-wide H3K4me3 patterns (representing PRDM9 binding). Chromosomal asynapsis was screened by the cytological analysis of spermatocytes [112]. A prominent feature of DSBs in these sterile hybrids was that each homologue (distinguishable by sequence differences) was targeted for DSBs at different chromosomal positions, with a preference of each PRDM9 allele towards the non-self chromosome, causing the so-called ‘asymmetric’ hotspot distribution [112]. Correlated to the asymmetry of PRDM9 targeting was the observation of delayed repair of DSBs, measured by the ‘heat’ of the DMC1 signal, and asynapsis of chromosomes leading to infertility [112]. An asymmetric distribution of PRDM9 targeting was also observed in other studies of hybrids of different crosses [75,76].

The main explanation for the asymmetric PRDM9 targeting and placement of DSBs observed in hybrids is motif disruption. Asymmetry occurs only in crosses of taxa that are evolutionary distant enough for the sequence erosion to be located only on the self-PRDM9, but not the foreign PRDM9 motifs, such that PRDM9 binds preferentially to the non-self-homologue resulting in each homologue having active hotspots at different genomic target sites [76,112].

However, hotspot asymmetry might not always occur in hybrid crosses, in spite of motif disruption. Hotspot asymmetry assumes that binding of one PRDM9 allele is independent of the binding of the other, with each PRDM9 allele acting as its own unit. However, this assumption might not always hold true. Baker *et al*. [121] demonstrated that PRDM9 forms a polymer with more than one PRDM9 unit and it is likely that PRDM9 acts within a polymer. Moreover, some PRDM9 alleles are dominant over others. In individuals with two different PRDM9 alleles, more DSBs or crossovers were found matching the motif recognized for one allele than the other, suggesting that one allele is dominant (e.g. in humans, PRDM9^C^ motifs were used over PRDM9^A^ in heterozygous individuals) [8,74]. Similar observations were made in hybrid mouse crosses or murine knock-ins, in which the binding motif for one of the PRDM9 allele was enriched (at different proportions depending on the cross) [5,75,76]. It is likely that this dominance is driven by a higher PRDM9 affinity to its target, but to date it is not known what factors influence the affinity of PRDM9 (type of ZnFs, clusters of specific ZnFs, length of the array, etc.).

In the case of dominance, the physical interaction between the two alleles in a multimer could result in the dominant allele masking the activity of the weak allele. In other words, in a polymer with different PRDM9 alleles, dominance might be amplified by the allele with the higher binding affinity driving the binding within the multimer to its recognition motif, and supressing at the same time the activity of the weaker allele trapped in the polymer [76,121]. Thus, hotspot asymmetry might be more likely to occur in crosses with PRDM9 alleles with similar dominance (e.g. as is the case for PWD and B6 [75]) than in crosses with strong differences in dominance, in which only one allele would be active, but not the other creating a more symmetric hotspot distribution. This hypothesis still needs to be proven; however, the observation that the absence of the second PRDM9 allele in a cross can partially re-establish symmetry as observed in PWD × B6 hybrids hemizygous or homozygous for only one PRDM9 allele (PRDM9^Pwd/-^ or PRDM9^Pwd/Pwd^, respectively) [112] is indicative of such a mechanism.

### (d) Hotspot asymmetry causes hybrid sterility

*Prdm9* has been called a speciation gene identified to be strongly associated with hybrid sterility [112,122]. Sterility in male hybrids between different crosses of murine subspecies has been repeatedly observed; however, offspring of crosses with different *Prdm9* alleles are not always sterile, but can be only partially sterile or even fertile [75,122–124]. Certain combinations of heterozygous PRDM9 alleles in some specific genetic backgrounds are incompatible [123,124]. For example, infertile male offspring were produced in the cross of two murine subspecies *M. m. musculus* and *M. m. domesticus* carrying two different *Prdm9* alleles [122,125]. The cellular mechanisms causing this sterility are not well understood, but primary spermatocytes of sterile PWD/Ph × C57BL/6 J (PWD × B6)F_1_ male hybrids showed asynapsis (lack of pairing) and mispairing (non-homologous synapsis of heterospecific homologues) of chromosomes at the pachytene stage. This asynapsis or mispairing resulted in apoptosis and abnormal spermatogenesis, and an arrested meiotic prophase due to the unrepaired recombination intermediates [122]. Both male and female meiosis showed a predisposition to asynapsis; however, certain genes enhanced or suppressed synapsis for divergent sequences between chromosomes in spermatogenesis, but not oogenesis [126]. The reciprocal cross C57BL/6 J × PWD/Ph (B6 female, PWD male) did not result in complete male F_1_ sterility [122,124].

It has been postulated that the preferential placement of DSBs on different genomic targets on each homologue (observed as asymmetric hotspots discussed previously) causes hybrid sterility [112]. This was shown in a very elegant experiment which exchanged the PRDM9-ZnF array by an evolutionary new allele (human B-allele) in a murine B6 × PWD and reciprocal cross that results in sterile male hybrids [112]. As expected, humanization of PRDM9 in a murine genetic background also changed the DSB hotspot landscape [112]. The humanized ZnF array targeted mainly DNA sequences enriched by the Myers recognition motif commonly found in human hotspots, and the original murine target motifs became silent for DSBs. The human ZnF array has evolved on a lineage separated from mice for approximately 100 Myr [105]. Hence, both heterozygous genomes of the murine hybrids were not eroded at the human PRDM9 target sites, and had therefore similar affinities for the newly introduced humanized ZnF array, and thus a symmetric distribution of DSBs on both homologues [112]. The effect of these newly specified hotspots was the rescue of fertility in the humanized F_1_ hybrids [112].

The reason of why asymmetric PRDM9 targeting causes a delayed repair of DSBs and sterility is still obscure, but it could be linked to a problematic strand exchange in regions with larger number of mismatches, although this hypothesis was considered unlikely [112]. Also, the involvement of the *Hstx2* locus located in the chromosome X containing genes associated with X-linked hybrid sterility might influence DSB repair genome-wide [127,128]. Alternatively, the dosage of PRDM9 and its binding sites might provide a plausible explanation. In the context of dosage, in a hybrid cross (e.g. PWD × B6) both the number of binding sites and PRDM9 alleles is half. Several studies have observed that the dosage of PRDM9 is an important determinant of numbers and activity of hotspots, as was described for hemizygous null mice (*Prdm9*^+/−^) with only one *Prdm9* copy. These mice had fewer numbers of hotspots that were also less active causing aberrant meiosis and reduced fertility [76]. It was also demonstrated that increasing the dosage of PRDM9, removing, or overexpressing a certain PRDM9 allele could rescue the fertility of completely sterile F_1_ hybrids [124], suggesting that the low dosage of some PRDM9 alleles may be insufficient to generate the minimal number of PRDM9-specified DSBs. Interestingly, dosage of PRDM9 does not determine the number of DSBs per cell, and PRDM9 murine knockouts, hemizygous null mice, as well as, murine hybrids have similar number of RAD51 and DMC1 foci (a proxy for DSBs) than wild-type mice [5,76,112]. A proposed explanation for this phenomenon is that as dosage of PRDM9 decreases, the default, PRDM9-independent hotspots become more active, but have trouble being repaired resulting in asynapsis [75].

Finally, the most parsimonious explanation given for the sterility in hybrids is that a symmetric distribution of hotspots (same genomic sites are targeted in both homologues) is needed for the proper homology search and nucleation of synapsis [112]. But why? A plausible reason might be that homology search requires an open chromatin stage present in both homologues for the proper homologue invasion and recognition, which might not be possible in DNA hidden within nucleosomes. An open chromatin stage is established by the H3K4me3 activity of the PRDM9 SET domain in PRDM9-dependent hotspots or by active promoters in PRDM9-independent hotspots (see next section). Note that imprinting (silencing of homologue-specific genes by DNA methylation at CpG islands (CGIs)) might also affect the symmetry of active promoters. However, further studies are needed to untangle the complex interplay between PRDM9 dosage, DSB symmetry, chromatin state and proper synapsis, that does not affect the number of DSBs, but strongly influence DSB repair and fertility.

## 5. Regulation of recombination by *cis*-factors acting at a local level

### (a) NDR, H3K4me3, transcription, CpG islands and sequence motifs

Other than the sequence recognition by PRDM9, there seems to be additional factors that influence hotspot activation. This is especially relevant for species without a functional PRDM9. The location of DSBs depend strongly on the base composition, chromatin accessibility, histone modifications or cohesion of the chromosomes (reviewed in [2,28]). As discussed in the previous section, DSBs are introduced at NDRs in chromatin loops tethered to the axis. Thus, chromosome accessibility at NDRs, together with mechanisms tethering these NDRs to the axis, is a major determinant of the placement of DSBs. NDRs are often found in promoter regions, upstream or at transcription start sites (TSS). Hence, hotspots are commonly also associated with promoters, promoter-containing intergenic regions flanked by divergent or tandemly oriented genes, or with promoter-like regions [4,129]. In fact, in *S. cerevisiae*, approximately 88% of all hotspots occur in promoter-associated NDRs [4,130,131]. However, not every NDR region is a DSB hotspot [4].

In *S. cerevisiae*, DSB formation is also influenced by H3K4me3 [35,36], which is an important chromatin modification at actively transcribed regions. Moreover, in *S. cerevisiae* strong DSB hotspots are located at 5′-ends in intergenic regions [4,132,133], where H3K4me3 sites are enriched and correlate with active transcription [36,134,135]. However, local transcription sites are not sufficient to establish a DSB, highlighting the importance of H3K4me3 [36]. H3K4me3 influences DSBs formation by driving the association of other proteins such as Spp1 that mediates indirect interactions between the chromosome axis and the H3K4me3 at the loop through its PHD finger [34,35]. SET1 is the catalytic subunit of the COMPASS complex responsible for all H3K4me3 marks in *S. cerevisiae* [34,36,135]. In the absence of SET1, the formation of DSBs is strongly reduced, while H3K4me3 sites are affected in a higher extent than dimethylated sites. Interestingly, in set1Δ mutants several new DSB sites appear suggesting that alternative DSB-forming pathways are activated in the absence of SET1 [36]. By contrast, in *S. pombe*, acetylation of the lysine 9 of histone H3 (H3K9ac) was prominent in more than 80% of recombination hotspots, but not H3K4me3 [136].

A strong predictor of hotspots is also transcription with recombination hotspots concentrating at promoter or promoter-like regions. Similarly to yeast, in the plant *A. thaliana* recombination correlates with TSS and TES (transcription stop sites), albeit with less intensity [137]. This is a common pattern also in other plants, with hotspots located in gene-rich regions (reviewed in [138]). In plants, the histone variant H2A.Z was also shown to be required for normal transcription and meiotic recombination, in addition to H3K4me3 [137]. Also in the two bird species, the zebra finch and long-tailed finch, recombination hotspots are strongly associated with actively transcribed TSS, TES and CGIs, supporting the model, that recombination is concentrated at functional elements with a poor nucleosome occupancy accessible to the recombination machinery. Interestingly, CGIs not associated with TSS and TES were found in 26% of hotspots in birds [53]. Elevation of recombination rates at TSS and CGIs (both considered a promoter feature) was also observed in swordtail fishes with a dysfunctional PRDM9 orthologue (without the KRAB and SSXRD domain and two missense substitutions in the SET domain) [52].

The role of CGIs in recombination is also prominent in dogs. In this species, hotspot regions at promoters are characterized by unmethylated CGIs, but not by H3K4me3 marks [54,139,140] (table 1). Also in *Arabidopsis* a high prevalence of hotspots at promoters with low DNA methylation was shown [137]. This observations direct to a possible link between absent PRDM9 and recombination directed to functional elements like TSS, TES or unmethylated CGIs which form an open chromatin stage and accessible for the recombination machinery [53].

**Table 1.** Factors that influence meiotic recombination in different taxa. For references see *H. sapiens:* (*a*) [8], (*b*) [43], (*c*) [141], (*i*) [24], (*j*) [23,142] (*k*) [5,45–47]; *M. musculus:* (*a*) [5], (*b*) [143], (c) [144], (*i*) [145], (k) [5,45–47]; *S. cerevisiae:* (*a*,*b*) [4], (*c*) [146], (*d*) [147,148], (*e*) [36], (*f*–*h*) [4,129], (*j*) [149,150]; *A. thaliana*:, (*b*) [151], (*c*) [152,153], (*e*) [137], (*f*) [137,154], (*g*) [154], (i) [137], (*j*) [137,155,156], (*l*) [137,157]; *D. melanogaster:* (*a*) [158], (*b*) [159–162], (*c*) [162,163], (*i*) [164]; *C. lupus familiaris:* (*a*) [139], (*c*) [165], (*d*) [139], (*f*, *i*) [139]; *T. guttata:* (*a*) [53], (*c*) [166], (*d*) [167], (*f*) [53]. Adapted from Cooper *et al.* [168].

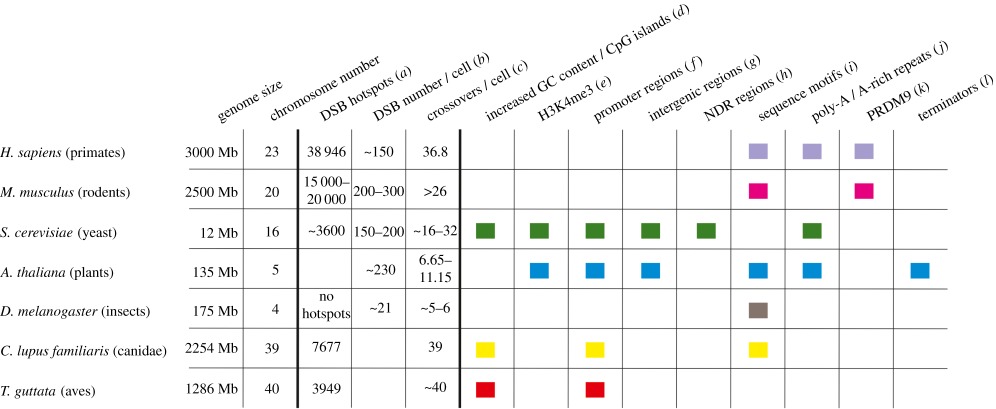

Certain sequence motifs also play a role in hotspot specification. There are sequence motifs which are naturally occurring NDRs based on their low intrinsic affinity to nucleosomes [169–171]. The best known are microsatellites that have been reported to alter the chromatin structure *in vitro* [172] and *in vivo* [173]. A mononucleotide microsatellite of five or more consecutive A's has the lowest reported nucleosome occupancy [169], as was also reported for poly (dA-dT) tracts *in vivo* and *in vitro* [170,171]. Interestingly, yeast hotspots are enriched with long poly-A runs with greater than or equal to 14 nt and short poly-G runs with 6–14 bp, but short poly-A tracts with 6–14 bp and long poly-G≥14 bp are under-represented [149]. Another study also showed a clear enrichment of poly-purine/poly-pyrimidine repeats, of which poly-A/T is a subset, in selected human and yeast hotspots [174]. In addition, poly-A tracts located upstream of TSS have been correlated with strong hotspots in plants [137,155], and a 14 bp poly-A tract plays a major role in the activity of the yeast *ARG4* hotspot [175].

In humans, there also seems to be a positive correlation of recombination with long poly-A tracts. In the analysis of recombination at a broad scale (with a resolution of 6 Mb), poly-A tracts between 4 and 6 As were found to be negatively correlated with recombination, but tracts greater than or equal to 8 As showed a significant positive correlation with recombination in humans and rats, but not mice [142]. An association of poly-A tracts and recombination at a fine scale was also reported for human hotspots (with a resolution in average of 5 kb), in which different repeat types were found more often in hotspots versus coldspots (including CT- and GC-rich repeats, as well as, homopolymers such as GAAAAAAAA and AAAAAAAAA) [23].

Thus, even in species with active PRDM9 these aforementioned *cis*-factors might play a role in hotspot specification and explain, for example, why different individuals showed at identical DNA regions (without polymorphisms) variation in recombination strength in spite of having the same PRDM9 allele [8].

### (b) What happens to hotspots in the absence of PRDM9?

The analysis of 227 vertebrates found that in more than half of the taxa some PRDM9 domains were missing (e.g. KRAB and SSXRD) [52]. It was also shown that these missing domains compromised the full functionality of PRDM9, and the recombination in these taxa (e.g. swordtail fish hybrids) was similar to the patterns observed in species lacking a functional PRDM9 such as dogs, zebra finch and long-tailed finch, and concentrated at TSSs and CGIs [52].

Usually, the lack of a functional PRDM9 does not compromise fertility. Taxa naturally lacking a functional PRDM9, like dogs (*Canis lupus familiaris*), chicken (*Gallus gallus*), frogs (*Xenopus tropicalis*) [51] and yeast are fertile and can also have concentrated recombination events in hotspots. It is different for a taxon in which PRDM9 was artificially removed like the murine *Prdm9* knock-out, which was infertile [5]. In these knockouts, DSBs are relocated to H3K4me3 regions in TSS of promoters [5]. Thus, PRDM9-mediated H3K4-trimethylation seems to play a role in placing hotspots far from functional genomic elements [5]. However, the relocation of DSBs in murine *Prdm9* knockouts also disrupts gametogenesis at the pachytene stage resulting in meiotic arrest due to asynapsis and impaired repair of DSBs [56]. It is plausible that the removal of PRDM9 is especially detrimental to fertility in species with prominent differences in imprinting, which could result in different stages of open chromatin at TSS sites between homologues. Interestingly, the number of DSB remains unchanged in *Prdm9* knockouts [57], suggesting that the density of DSB is tightly regulated.

Recently, a study reported about a healthy, fertile *Prdm9* knock-out human mother harbouring a mutation in the PR/SET domain causing a truncated version of the protein lacking the ZnF domain. In this individual, only a minor percentage of the 39 crossovers occurred within PRDM9-dependent hotspots or historical hotspots characterized in the human genome. Moreover, unlike other PRDM9-inactive taxa, crossovers in this knock-out mother were not enriched at promoter or promoter-like regions with increased GC content [176].

In spite of the sequence erosion occurring at recombination hotspots, organisms without PRDM9, like yeast and birds, show against all odds a high conservation of hotspots over several millions of years, in terms of strength and hotspot location [53,177]. In birds, gBGC is a strong driver of sequence evolution at hotspots increasing the GC content in these regions and also genome wide, particularly in the PAR region [53]. Yet, this increase in GC content does not seem to affect the local hotspot [53] or the broad-scale (10–1000 kb) recombination landscape in bird lineages separated by millions of years [53 1575]. This suggests that recombination in PRDM9-free species is controlled by several layers including the structural architecture of the DNA during meiotic prophase I, which is very stable likely due to selectively constrained features [177].

The high degree of hotspot conservation in species without PRDM9 restricted to NDRs at gene promoters as documented in birds [53] and yeast [177], poses the question whether this has an impact in the overall number of hotspots and their strength. It seems that species expressing an active PRDM9 exhibit more hotspots than species without PRDM9 (table 1). For example, the average distance between hotspots in PRDM9 active species, like primates and mice, is between 50 and 140 kb, whereas, this distance increases to approximately 300–320 kb in dogs and birds, respectively. A possible explanation could be that the definition of a hotspot is more ambiguous, differs between studies or the power to detect hotspots is also highly variable. Alternatively, it is possible that in species lacking PRDM9, the recombination ranges over broader genomic regions and is less concentrated in discrete hotspots. In fact, hotspots in plants are on average approximately 2.5 kb but could be as large as 23 kb (reviewed in [138]). An exception is yeast with a DSB hotspot every 3 kb, but this could be due to its comparatively gene-dense genome.

## 6. Structural DNA features in recombination hotspots acting at a large scale (potentially independent of local factors)

### (a) DNA methylation

As discussed in the previous section, CGIs are usually located upstream of genes and have been correlated with the local recombination activity depending on their methylation state (table 1). However, DNA methylation could also be acting at a larger scale and explain differences in the recombination landscape. It was observed that genome-wide recombination covaries with methylation levels in humans [178]. However, in another study, it was observed in the analysis of the sperm methylome that methylation correlates with recombination only at larger genomic scales (500 kb windows) in humans and in chimpanzees, especially in repeat regions, but hotspots correlated only weakly with methylation at a fine scale (several kb) [179].

Evidence for a role of DNA methylation in human hotspot activity comes initially from imprinted DNA regions. In humans, imprinted regions with different methylation levels have pronounced differences in recombination activity between the sexes (reviewed in [9]). In addition, the recombination landscape is altogether different between human males and females. Human recombination maps based on pedigree analysis, the immunofluorescent localization of DMC1 foci, and the comparison of male DSB maps with historical maps showed more subtelomeric recombination in males than in females, and an overall higher number of recombination events in females [8,18,19,180]. Moreover, using pedigrees to map crossovers it was observed that the genetic map in males is shorter than in females [18,19]. Differences in recombination between the two sexes was also observed in mouse within a subtelomeric region, where a 27Mb telomeric segment in a cross between C57BL/6 J and CAST/EiJ strains (B6xCst) showed differences in recombination rates between males and females [181].

Sex differences in recombination are widespread in mammals, but the causes of this pattern are poorly understood. The cytological analysis of oocytes and spermatocytes showed that male/female differences in recombination are established at the formation of DSBs showing strong differences in the length of the synaptonemal complex and DNA loop size [182]. The difference in the recombination landscape, in addition to differences in chromosome compaction, might also be explained by the DNA methylation of primary spermatocytes and oocytes. The male germline is highly methylated, especially in repeat regions containing transposable elements (reviewed in [183]). In humans, the first reductional division (Meiosis I) occurs in the adult stage with fully methylated genomic DNA; whereas, in females, this division takes place in the fetus when genomic DNA is only partially methylated (reviewed in [184]).

Congruent with methylation acting on a larger scale, it seems that CpG methylation drives recombination independently of the activity of PRDM9. This hypothesis might explain the observation made in a genetic study that revealed that humans and chimpanzees have mainly different recombination landscapes (expected given the differences in the PRDM9-ZnF array), except around genes and CGIs [50]. This suggests that similarities around specific genomic features could, complementary or independently of PRDM9, play a role in specifying recombination [50]. However, to date the role of large-scale factors and their contribution in the recombination landscape is still unclear.

Further evidence that methylation affects recombination at a larger scale also comes from other organisms without PRDM9. It has been observed that in the fungus *Ascobolus immersus*, DNA methylation strongly inhibits CO formation [185]. Similarly, in *Arabidopsis*, it was shown that methylation silences hotspots and controls the chromosomal domains undergoing recombination [186]. In addition, in this plant species crossovers were suppressed in hyper-methylated regions [187], and hypo-methylation increased CO rates in euchromatic regions [188].

The role of methylation in recombination is not yet fully understood (also reviewed in [179]), but it is likely that DNA methylation is directly associated to chromatin remodelling and DNA accessibility, especially in species without PRDM9. It is possible that DNA methylation plays a role in the formation of NDRs at a large scale, and to a lesser degree directly affects the binding of *trans*-factors like PRDM9 or CXXC1, at a fine scale [66].

### (b) Chromosomal effects

The physical location of DSBs along the chromosome is also a rather important parameter in the recombination landscape. For example, it was shown in yeast that DSB formation is less frequent around centromeres and telomeres than in central regions of the chromosome arms [4,189,190]. It is likely that axis proteins are the main players determining the distribution of DSBs along the chromosome. The axis protein REC8 is required for the early localization of SPO11, and rec8Δ mutants reduce DSB formation [191] in a chromosome domain-specific fashion. REC8 correlates with domains requiring the recruitment of the DSB machinery to the chromosome axis [33]. REC8 has been shown to recruit directly the axis components Red1 and Hop1, which in turn recruit and activate the DSB machinery [33,132]. In some places, Red1 and Hop1 bind to chromatin independently of REC8, and are able to promote DSBs at normal levels at their binding sites, explaining the locally varying effects of rec8Δ on recombination [191]. A very intriguing regulation occurs in the sex chromosomes that follow slightly different rules in male meiosis, because their pairing is restricted to the very small PAR regions and the DSB repair is delayed until the autosomes have been almost fully processed. In order to ensure successful pairing, PAR has three- to sevenfold shorter loops and exhibits an about 20-fold higher recombination rate per kilobase [192]. Additionally, the axis length relative to the DNA content is increased by 10-fold. Two recent studies have identified that non-PAR regions of the sex chromosomes are also prone for DSBs at gene promoter regions, but at lower levels [7,75].

Chromosome size also plays a major role in the number of DSBs. Smaller chromosomes have more difficulties to find their homologues, and thus require a higher DSB density than larger ones (reviewed in [193]). The minimal number of DSBs necessary for a successful chromosome pairing is still ambiguous, but it seems to be conserved even among divergent taxa regardless of the genome size (table 1). For example, yeast genomes experience about 160 DSBs per cell and mice 230–400 per spermatocyte, with variations between males and females (reviewed in [1]); although, their genome differs by two orders of magnitude. The plasticity in the number of DSBs that a genome can tolerate might vary between organisms, but it is important to control the total number of DSBs to avoid too few or too many DSBs. Several organisms like mice, flies and yeast have developed a feedback mechanism regulating the number of DSBs in a cell. ATM and ATR kinases are activated after the DSB induction and lead to post-translational modifications and downregulation of DSB-forming accessory proteins like REC114 which activates SPO11, thereby decreasing SPO11 activity [7,194]. Not only the total number of DSBs per chromosome, but also the location and distance to neighbouring DSBs is regulated, in part, by the ATM kinase [195]. ATM and ATR kinases operate in *cis*, as well as in *trans* to determine the DSB formation over the whole genome [195,196]. The exact mechanisms of the *cis* regulation by the ATM kinase are not resolved yet, but it is possible that ATM masks neighbouring upstream and downstream sequences to ensure the introduction of only one DSB in a primed hotspot within a loop creating an interference zone of approximately 70–100 kb [168].

### (c) Conclusion

DSBs, and thus recombination rates at individual hotspots vary over orders of magnitude within and between species. The location and relative activity of hotspots is regulated in some species by DNA sequences (motifs) recognized by the *trans*-acting factor PRDM9. An intrinsic property of recombination is the sequence erosion due to the mutagenic activity of recombination and BGC. This sequence erosion at motifs leads to a weaker PRDM9 binding and hence hotspot activity, which is recovered by the rapid evolution of PRDM9 that binds new targets. In hybrid species, the placement of DSBs is biased to the less eroded chromosome homologue causing in some cases an asymmetric distribution of DSB hotspots, mispairing and sterility. In spite of the sequence erosion at hotspots, taxa without an active PRDM9 show an astonishing stability of hotspots in strength and location over millions of years, suggesting that selectively constrained structural features likely controlled by epigenetic modifications or chromosomal features are highly conserved and are the main determinants of recombination. The further study of DSB distribution, control and conservation, as well as the relationship of DSB placement and its regulation (e.g. interference) will provide important insights into this tightly regulated process, as well as its evolutionary consequences, and will identify further biological key players of this very complex process.

## 7. Material and methods

### (a) Experimental set-up

In order to determine the effect of DNA methylation on the binding specificity of the murine PRDM9^Cst^-ZnF domain to DNA of the murine *Hlx1^B6^* hotspot, competition assays were performed using EMSA. Therefore, the binding of the PRDM9^Cst^-ZnF domain to a biotin-labelled 75 bp DNA fragment (referred to as hot DNA) of the *Hlx1^B6^* hotspot was recorded in a series of nine binding reactions by additionally adding an increasing amount of a certain 39 bp unlabelled DNA fragment (referred to as cold DNA). PRDM9-bound DNA migrates slower in the gel as compared to free DNA and is therefore visible as a shifted band. The cold fragment differs in each experiment and was titrated from 0- to 100-fold excess according to the concentration of the hot fragment and therefore competes for PRDM9-ZnF binding. With increasing amount of the cold DNA, the shifted band decreases at different rates depending on the sequence specificity.

### (b) Production of the DNA fragments

#### (i) Hot DNA

To standardize quality of PCR amplicons, the hot 75 bp *Hlx1*^B6^ DNA fragment was produced in two successive PCR reactions using the biotin-labelled primers Bio-Hlx1–75bp_F and Bio-Hlx1–75bp_R (primer sequences are shown in table 2) as it was described in [63]. In the first PCR round, 1 ng µl^−1^ genomic DNA of the mouse strain C57BL/6 J (B6) was used as starting template (kindly provided by the Pektov Lab, Center for Genome Dynamics, The Jackson Laboratory, Bar Harbor, ME 04609, USA). As polymerase, 0.75 units/50 µl of the OneTaq Hot Start DNA polymerase (NEB) was used in 1X OneTaq Standard Reaction Buffer (20 mM Tris–HCl, 22 mM NH_4_Cl, 22 mM KCl, 1.8 mM MgCl_2_, 0.06% IGEPAL CA-630, 0.05% Tween 20, pH 8.9 at 25°C) supplemented with 200 µM dNTPs (Biozym) in a total reaction volume of 50 µl. The correct length of the amplicon was assessed via gel electrophoresis. In order to get rid of single-stranded DNA molecules and primers, an Exonuclease I digest was performed followed by a purification using the Wizard SV Gel and PCR Clean-Up System (Promega) according to manufacturer's instructions. The concentration of the pure DNA fragment was determined using a Nanodrop 2000 instrument (Thermo Scientific). For more details, see [63].

**Table 2.** Overview of single-stranded oligonucleotide sequences used in this study. Shown are biotin-labelled (Bio) primer sequences used for amplifying the 75 bp DNA fragment of the murine *Hlx1*^B6^ hotspot as well as single-stranded synthetic fragments that were hybridized to create double-stranded DNA fragments with different methylation levels. Red bold letters indicate the positions of the 5-methyl-cytosines.

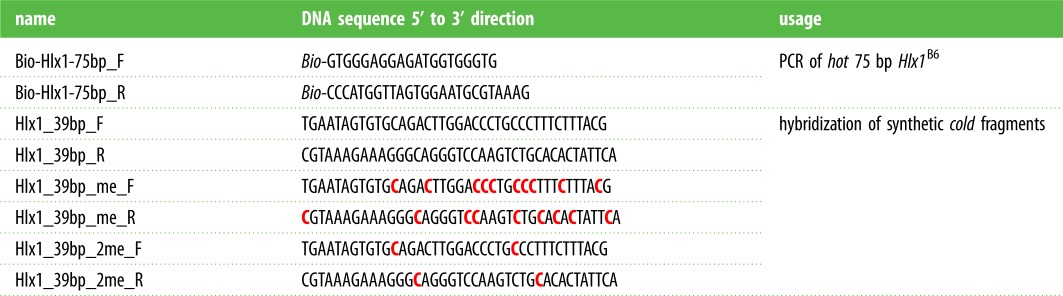

#### (ii) Cold DNA

The cold or unlabelled 39 bp *Hlx1*^B6^ DNA fragments were ordered as lyophilized, HPSF purified single-stranded synthetic complementary oligonucleotides at the company Eurofins. Methylated targets were ordered containing 5-methyl-cytosines at selected positions of the DNA fragments (see oligonucleotide sequences in table 2). The single-stranded oligonucleotides were resuspended in hybridization buffer (10 mM Tris, 50 mM KCl, 1 mM DTT, pH 7.5) and equal amounts of forward and reverse strands were mixed and hybridized, starting with 3 min at 98°C following with a temperature decrease of 1°C min^−1^ to form double-stranded DNA fragments. Exonuclease I digest and purification of the hybridized products was performed as described in [63].

### (c) Cloning and expression of YFP-PRDM9Cst-ZnF

The sequence of the Exon 10 of the *Prdm9^Cst^* gene, encoding the ZnF domain of the protein, was cloned in the pOPIN-M vector system, containing the maltose-binding protein (MBP) for enhanced solubility, using the Gibson AssemblyTM cloning kit (NEB) resulting in a His-MBP-eYFP-PRDM9^Cst^-ZnF fusion construct (123 kDa) that was bacterially expressed using the *Escherichia coli* strain Rosetta^TM2^(DE3)pLacI (Novagen, Merck). As lysate buffer we used 1xTBS (25 mM Tris base, 137 mM NaCl, 2.7 mM KCl, pH 7.4) supplemented with 0.3% Sarcosyl (*N*-Lauroylsarcosine) to create a whole-cell protein lysate (WC*), as described in [63]. The PRDM9 concentration was estimated to be 49.31 µM by a Capillary Western [63]. For more detailed description of cloning, expression and lysate preparation, see [63].

### (d) Binding reactions

#### (i) EMSA reaction

The EMSA binding reaction was performed using the following buffer conditions: 10 mM Tris–HCl pH 7.5, 50 mM KCl, 1 mM DTT, 50 ng µl^−1^ polydIdC, 0.05% NP-40 and 50 µM ZnCl_2_. The binding components of 15 nM hot DNA, 0–1500 nM cold DNA and 250 nM of His-MBP-eYFP-PRDM9^Cst^-ZnF protein whole-cell lysate in 1×TBS+0.3% Sarcosyl were added simultaneously to the binding reaction and incubated for 1 h at RT. In each experiment one reaction with only the biotin-labelled DNA and one reaction without the cold DNA (referred to as reference band) was performed. Binding reactions were then separated on a 5% polyacrylamide gel for 45 min at 100 V during electrophoresis. The EMSA protocol was carried out as it was described in [63]. All experiments using different cold DNA fragments were performed at least in triplicates.

#### (ii) Image analysis

Images with exposure times of 1 s and *γ* values of 0.5 were used for analysis with the Image Lab software (Bio-Rad). The intensities of the shifted bands were measured and the relation of each band to the reference band without the addition of cold DNA was calculated (referred to as relative intensity). Using OriginPro8.5, the relative intensities were plotted against the increasing concentration of the cold competitor in a semi-logarithmic graph and fitted with an exponential function (ExpDec1).

#### (iii) Statistical analysis

We tested differences in binding trends with a generalized least square model using a likelihood ratio test that takes non-homogeneous variances and auto-correlation into account. Detailed descriptions are found in [63].

## Data accessibility

This article has no additional data.
